# Affinity-Controlled Double-Network Hydrogel Facilitates Long-Term Release of Anti-Human Papillomavirus Protein

**DOI:** 10.3390/biomedicines9101298

**Published:** 2021-09-23

**Authors:** Chenjia Zhao, Jingyuan Ji, Tianjun Yin, Jing Yang, Yuan Pang, Wei Sun

**Affiliations:** 1 Biomanufacturing Center, Department of Mechanical Engineering, Tsinghua University, Beijing 100084, China; zhaocj16@mails.tsinghua.edu.cn (C.Z.); jijy18@mails.tsinghua.edu.cn (J.J.); yintj330@163.com (T.Y.); 2 Biomanufacturing and Rapid Forming Technology Key Laboratory of Beijing, Beijing 100084, China; 3 Overseas Expertise Introduction Center for Discipline Innovation, Tsinghua University, Beijing 100084, China; 4 Key Laboratory for Advanced Materials Processing Technology, Ministry of Education, Beijing 100084, China; 5 Division of Regenerative Medicine and Cellular Therapies, School of Pharmacy, University of Nottingham, Nottingham NG7 2RD, UK; Jing.Yang@nottingham.ac.uk; 6 Department of Mechanical Engineering, Drexel University, Philadelphia, PA 19104, USA

**Keywords:** double-network hydrogel, affinity-controlled release, cervix implant, anti-HPV, DLP printing

## Abstract

Hydrogels have recently received attention as delivery carriers owing to their good biocompatibility and structural similarity to natural extracellular matrices. However, the utilization of traditional single-network (SN) hydrogels is limited by poor mechanical properties and burst drug release. Therefore, we developed a novel double-network (DN) hydrogel, which employs an alginate (ALG)/polyethylene glycol diacrylate (PEGDA) network to adjust the mechanical strength and a positively charged monomer AETAC (2-(acryloyloxy)ethyl]trimethyl-ammonium chloride) to regulate the release curve of the electronegative anti-human papillomavirus (HPV) protein (bovine β-lactoglobulin modified with 3-hydroxyphthalic anhydride) based on an affinity-controlled delivery mechanism. The results show that the double-network hydrogel strongly inhibits the burst release, and the burst release amount is about one-third of that of the single-network hydrogel. By changing the concentration of the photoinitiator, the mechanical strength of the DN hydrogels can be adjusted to meet the stiffness requirements for various tissues within the range of 0.71 kPa to 10.30 kPa. Compared with the SN hydrogels, the DN hydrogels exhibit almost twice the mechanical strength and have smaller micropores. Cytotoxicity tests indicated that these SN and DN hydrogels were not cytotoxic with the result of over 100% relative proliferation rate of the HUVECs. Furthermore, DN hydrogels can significantly alleviate the burst release of antiviral proteins and prolong the release time to more than 14 days. Finally, we utilized digital light processing (DLP) technology to verify the printability of the DN hydrogel. Our study indicates that ALG/PEGDA-AETAC DN hydrogels could serve as platforms for delivering proteins and show promise for diverse tissue engineering applications.

## 1. Introduction

In recent years, advances in biotechnology and genetic engineering technologies have resulted in the development of a variety of therapeutic recombinant proteins. The bioengineered protein drug market is one of the fastest growing areas in the pharmaceutical and biotechnology markets [1]. These drugs have received extensive attention from pharmaceutical companies because of their biological reactivity, specificity, safety, and overall success rate in treating diseases [2,3,4]. The global market for bioengineered protein drugs is expected to reach $228.4 billion by 2021 from $172.5 billion in 2016 [5]. However, the basic dosage form of therapeutic proteins is a lyophilized agent, and its efficacy is limited owing to its low absorption efficiency and short biological halflife. Therefore, frequent injections are usually required in the long term, which in turn increases the risk of adverse side effects associated with overdose [6]. Researchers have invested effort into developing drug delivery systems that can control the release and improve the efficacy of protein and peptide drugs, reduce adverse reactions, and improve the quality of life of the patients [7,8,9].

So far, many delivery systems based on organic or inorganic materials have been developed to achieve effective and sustained delivery of therapeutic agents [10,11,12,13]. Hydrogels are three-dimensional networks of crosslinked hydrophilic polymers. Owing to their biocompatibility, ease of preparation, and unique physical properties (such as adjustable porosity and affinity for biological fluids), hydrogels play an important role in overcoming the limitations of current delivery systems. The mesh size and hydrophilicity of the hydrogel matrix can be adjusted by controlling the crosslinking density to provide adjustable protein release properties. However, traditional single-network (SN) hydrogels generally do not meet the requirements of carriers for delivery applications [14]. One of the main problems of SN hydrogels is that they have a single composition, which has a negative impact on the ability of the hydrogel to regulate protein release. In addition, the mechanical properties of SN hydrogels are poor, mainly owing to their uneven brittle network, which easily breaks after the application of force. These factors limit the applicability of SN hydrogels as sustained release carriers for therapeutic molecules [15]. Double-network (DN) hydrogels combine two different networks that have a synergistic effect on the physical and mechanical properties [16,17]. One of the most prominent features of DN hydrogels is their excellent mechanical properties, including high toughness, destruction resistance, and high water content [18]. Compared with SN hydrogels, these advantages make them better candidates for new protein delivery platforms [19].

Sodium alginate is a natural polysaccharide obtained from seaweeds and bacterial sources. It consists of hexuronic acid residues, β-D-mannuronic acid (M), and α-L-guluronic acid (G). It is readily crosslinked with cations such as Ca^2+^ (ionic crosslinking) [20] and is a popular biomaterial owing to its biocompatibility, biodegradability, durability, injectability, and ability to form a 3D scaffold [21]. Immobilization of growth factors such as bone morphogenetic protein 2 (BMP-2) and vascular endothelial growth factor (VEGF) in alginate delivery systems offers various applications in tissue engineering [22,23]. Moreover, the combination of alginate with other natural biomaterials, such as collagen and hyaluronic acid, makes this composite promising for application in nerve and cardiac tissue engineering [24,25]. Polyethylene glycol (PEG) is an inert hydrophilic polymer that consists of polyether groups, which exhibit strong hydrogen bonds with water molecules [10]. PEG is available in different geometries and hydrophilicities and can be coupled to hydrophobic moieties to produce a nonionic surfactant. The clinical viability of PEG is increased by surface modification with several functional groups, such as RGD, mono, or di acrylate groups (crosslinkable by UV light) [10]. Cai et al. [26] used polyethylene glycol diacrylate (PEGDA) to cocrosslink with thiolated hyaluronic acid and chondroitin sulfate, and the synthesized hydrogel released basic fibroblast growth factor (bFGF) and promoted wound healing.

In addition to regulating material degradation or crosslinking density to control protein release, adjusting the interaction between the protein and the material is very effective [27]. The affinity-based drug delivery system was originally inspired by the controlled release mechanism found in the extracellular matrix (ECM), such as the binding of heparin to a variety of proteins [28]. These systems use transient interactions between proteins and binding ligands to slow the diffusion and release of therapeutic agents from the hydrogel network. Affinity-controlled systems are attractive for the delivery of therapeutic proteins [29]. Based on the affinity-controlled release mechanism, in this study we developed a DN hydrogel system, which employs an ALG/PEGDA network to adjust the mechanical strength and the positively charged monomer 2-(acryloyloxy)ethyl]trimethyl-ammonium chloride (AETAC) solution to adjust the release curve of the electronegative anti-human papillomavirus (HPV) protein (bovine β-lactoglobulin modified with 3-hydroxyphthalic anhydride) [30,31,32]. Due to a lack of delivery system and high solubility of 3-hydroxyphthalic anhydride-modified bovine beta-lactoglobulin [30], existing studies can only culture cells directly in a protein-containing solution [31]. As for the in vivo application, the previous clinical application method was to use it in a gel, but it needs to be applied once every two days, which is inconvenient for patients [33]. Therefore, the hydrogel system with controlled release function in this study has great application prospects. Compared with the PEGDA SN hydrogel, the mechanical strength of the ALG/PEGDA-AETAC DN hydrogel was higher and could be adjusted to values between 0.7 and 10.3 kPa. At the same time, DN hydrogels significantly alleviated the burst release of antiviral proteins and prolonged the release time to >14 days. Combined with the biomimetic cervical reconstruction model designed in our previous research, digital light processing (DLP) technology was used to print the DN hydrogel to construct drug-loaded cervical biomimetic implants [34]. We expect that our hydrogel matrix, which allows for adjustable mechanical strength and controlled long-term delivery profiles, will broaden the applications of biomedical materials.

## 2. Materials and Methods

### 2.1. Materials and Cell Preparation

Anti-HPV protein (bovine β-lactoglobulin modified with 3-hydroxyphthalic anhydride) was supplied by Jingbo Biological Pharmaceutical Co., Ltd. (Taiyuan, China). A stock solution was prepared in deionized (DI) water at a concentration of 26.9 mg/mL. 2-Hydroxy-4-(2-hydroxyethoxy)-2-methylpropiophenone (Irgacure 2959) and [2-(acryloyloxy)ethyl]trimethylammonium chloride solution (AETAC) were purchased from Sigma-Aldrich (St. Louis, MO, USA). Sodium alginate (ALG, (C_6_H_9_O_7_Na)_n_, Mn = 200,000, G blocks: M blocks = 39:61, low viscosity) was obtained from JiSiEnBei International Trade Co., Ltd. (Hongkong, China). Polyethylene glycol diacrylate (PGEDA, Mn = 6 kDa) was purchased from Huateng Pharmaceutical Co., Ltd. (Changsha, China). NIH 3T3 cells (American Type Culture Collection, Manassas, VA, USA) and human umbilical vein endothelial cells (HUVECs, China Infrastructure of Cell Line Resources, Beijing, China) were cultured in high-glucose Dulbecco’s modified Eagle’s medium (DMEM) (Invitrogen, Grand Island, NY, USA) supplemented with 10% fetal bovine serum (FBS, Hyclone, Grand Island, NY, USA), 1% nonessential amino acid solution (NEAA, Gibco, Grand Island, NY, USA), 1% GlutaMAXTM-I (Gibco, Grand Island, NY, USA), and 1% antibiotics (penicillin and streptomycin, Gibco, Grand Island, NY, USA) at 37 °C in a humidified atmosphere containing 5% CO_2_.

### 2.2. Preparation of Hydrogels

#### 2.2.1. Preparation of PEGDA-AETAC SN Hydrogels

PEGDA-AETAC SN hydrogels were formed by free radical polymerization of PEGDA and [2-(acryloyloxy)ethyl]trimethyl-ammonium chloride solution (AETAC), an electropositive monomer, in DI water and filtered using microporous membrane filters with 0.22 μm pore size. The wt% of PEGDA and AETAC was 20% and 5%, respectively. The photoinitiator Irgacure 2959 (0.1%, 0.2%, and 0.4% *w*/*v*) was added to the polymeric mixtures before UV irradiation. Then, 500 μL aliquots were transferred to sterile silastic molds (15 × 15 × 5 mm) and photoinitiated under UV light exposure (365 nm, ~2.6 mW cm^−2^, UVP XX-15BLB, Analytikjena, Upland, CA, USA) for 5 min.

#### 2.2.2. Preparation of ALG/PEGDA-AETAC DN Hydrogels

**Figure 1:**
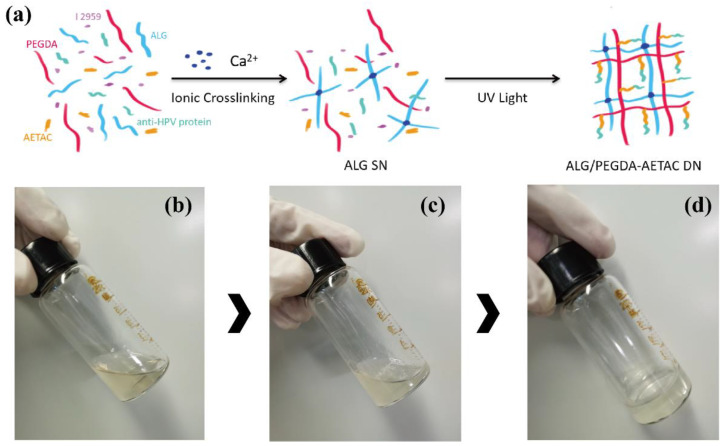
(**a**) Schematic diagram of the formation of ALG/PEGDA-AETAC DN hydrogels. (**b**) ALG/PEGDA-AETAC mixture solution. (**c**) Formation of the first network, ion-crosslinked ALG-Ca. (**d**) Formation of the second network, photocrosslinked PEGDA-AETAC.

DN hydrogels were crosslinked according to a previously published method [35]. PEGDA and AETAC were dissolved in deionized (DI) water (40 wt% and 10 wt%, respectively) and mixed with sodium alginate solution in water (5 wt%) in a volume ratio of 1:1. Irgacure 2959 (0.1%, 0.2%, 0.4% *w*/*v*) and calcium chloride (25 µL of 1 M CaCl_2_ per mL of pre-gel solution) were added to the polymeric mixtures as photoinitiators for PEGDA and ionic crosslinkers for alginate, respectively, followed by filtration with microporous membrane filters with 0.22 μm pore size. Then, 500 μL aliquots were transferred to sterile silastic molds (15 × 15 × 5 mm) and photoinitiated under UV light exposure (365 nm, ~2.6 mW cm^−2^, UVP XX-15BLB, Analytikjena, Upland, CA, USA) for 5 min. A schematic of the entire process is shown in Figure 1a.

### 2.3. Characterization of Hydrogels

#### 2.3.1. Mechanical Properties

The compressive properties of PEGDA-AETAC SN hydrogels and ALG/PEGDA-AETAC DN hydrogels with different wt% of Irgacure 2959 were tested using an Endura TEC ELF 3200 (Bose). The samples were 16 × 16 × 3.5 mm. A force transducer (±50 N) was utilized in an unconstrained uniaxial compression test at a loading rate of 0.5 mm/s, a data acquisition rate of 4 s^−1^, and a maximum compression distance of 10% of the sample height.

Based on the mechanical testing results, the concentration of Irgacure 2959 was fixed at 0.2% for the subsequent experiments, and the SN and DN hydrogels were prepared according to the method described in Section 2.2.

#### 2.3.2. Scanning Electron Microscopy

The prepared hydrogels were frozen at −20 °C for 24 h, followed by lyophilization in an Alpha 1−2 freeze dryer (Martin Christ GmbH, Osterode am Harz, Germany) for 48 h. Afterward, the freeze-dried hydrogels were cut using scissors. The morphology of the hydrogel crosssections was observed using scanning electron microscopy (SEM) (ZEISS, GeminiSEM 300, Oberkochen, Germany) with a working voltage of 5 kV. All specimens were sputter-coated with gold-palladium (50 nm).

#### 2.3.3. Swelling Ratio Test

The swelling properties of the hydrogels were examined by monitoring the weight of the hydrogel. PEGDA-AETAC SN hydrogels and ALG/PEGDA-AETAC DN hydrogels were soaked in PBS at 37.0 °C ± 0.1 °C. At each preset time, they were removed, and the surface moisture was wiped off using tissue paper. The hydrogels were weighed as soon as possible and then returned to the original PBS. The swelling ratio (SR, %) was calculated using Equation (1):

(1) $$\mathrm{SR}=\frac{W_{t}-W_{0}}{W_{0}}\times 100\%$$

where $W_{t}$ and $W_{0}$ are the weights of the hydrogels at time t and zero, respectively.

#### 2.3.4. Cytotoxicity Testing of Hydrogels

The polymerized PEGDA-AETAC SN hydrogels and ALG/PEGDA-AETAC DN hydrogels were soaked in PBS for 4 h to remove the unreacted reagents. According to the ISO Standard 10993-12, the 5 mL complete culture medium was incubated with the SN and DN hydrogel pieces at 37 °C in 5% CO_2_ for 24 h, and the extraction ratio was 0.1 g/mL. NIH-3T3 cells and HUVECs were respectively seeded at a density of 1.5 × 10^4^ cells/well in 24-well plate for 12 h, followed by culturing in the extract medium for two days. NIH-3T3 cells and HUVECs cultured in normal medium were used as controls, and the cell-free culture medium was used as the blank sample. When incubated at 24 and 48 h, cellular proliferation in each group was evaluated using the Cell Counting Kit-8 (CCK-8 kit, Dojindo Molecular Technologies, Inc., Kumamoto, Japan) for the characterization of cytotoxicity. The absorbance of the untreated group was determined as a negative control (100%), and the relative cell proliferation of the treated group was determined according to Equation (2).

(2) $$\mathrm{Relative}\;\mathrm{cell}\;\mathrm{proliferation}=\frac{OD_{treated}-OD_{blank}}{OD_{control}-OD_{blank}}\times 100\%$$

NIH-3T3 cells and HUVECs were treated with calcein AM (Sigma-Aldrich, St. Louis, MO, USA) and propidium iodide (Sigma-Aldrich, St. Louis, MO, USA) solution for 30 min to stain live and dead cells, respectively. Fluorescent images were captured using a laser-scanning confocal microscope (LSCM; LSM 710 META, Zeiss, Oberkochen, Germany).

### 2.4. Protein Loading and Releasing

The solutions were prepared and filtered as shown in Table 1. The choice of protein concentration was based on clinical dosages. Aliquots without anti-HPV proteins were also prepared as control groups. Then, 500 μL of the mixture was transferred to sterile silastic molds (15 × 15 × 5 mm) and photoinitiated under UV light exposure (365 nm, ~2.6 mW cm^−2^, UVP XX-15BLB, Analytikjena, Upland, CA, USA) for 5 min. Afterward, the prepared hydrogel pieces were suspended in 5 mL of PBS at 37 °C. Releasing buffers were collected at each preset time point and replaced with fresh PBS. The concentration of the anti-HPV protein was quantified using a detergent-compatible Bradford assay kit. The cumulative release of proteins from the hydrogels was calculated using Equation (3):

(3) $$R_{n}=\frac{V_{e}\sum_{k=1}^{n}C_{k}}{W_{total}}\times 100\%$$

where $R_{n}$ is the cumulative release of protein, $V_{e}$ is the volume of each sample for analysis, $C_{k}$ is the protein concentration in the release solution at the kth sampling, and $W_{total}$ is the quantity of protein loaded on the hydrogel.

### 2.5. 3D Printing of Cervix Implant

In view of the fact that the ALG/PEGDA-AETAC mixture was still liquid after ion crosslinking, DLP (digital light processing) technology was applied to print the DN hydrogels. A model of the word “THU” was constructed by Solidworks 2018 (Dassault Systèmes, Waltham, MA, USA), and each letter was 1.5 mm in length and width and 4 mm in height. Based on our previously published study, the cervical implant was designed as a conical structure 30 mm in diameter at its base and 10 mm in height, with a hollow channel of 5 mm in diameter vertically penetrating the model, which was constructed according to the excision shape in cervical cancer conization surgery [30]. The word model and cervix model were sliced using Photon WorkShop V2.1.26 (Anycubic, Shenzhen, China). After that, they were printed and molded in Photon Mono, applying a layer thickness of 100 μm and an exposure time of 3 s per layer. 

### 2.6. Statistical Analysis

The researchers conducting the tests described above were blinded to the identity of each sample. T-tests measuring means ± standard deviation of each group of data were compared using SPSS software (version 11.0, IBM, Armonk, NY, USA). Differences between results were analyzed using the Mann-Whitney test and accepted as significant at *p* ≤ 0.05.

## 3. Results and Discussion

### 3.1. Preparation of ALG/PEGDA-AETAC DN Hydrogel

In this work, DN hydrogels were synthesized using a two-step method, as shown in Figure 1a. Sodium alginate and PEGDA were soluble in ultrapure water (Figure 1b). Positively charged calcium ions were complexed with the negatively charged alginate to form the first network, while the mixture solution became a more viscous liquid (Figure 1c). After irradiation with ultraviolet light, a radical polymerization reaction occurred under the induction of the photoinitiator to construct the second network, resulting in the formation of the DN hydrogel (Figure 1d).

### 3.2. Characterization of the SN and DN Hydrogels

**Figure 2:**
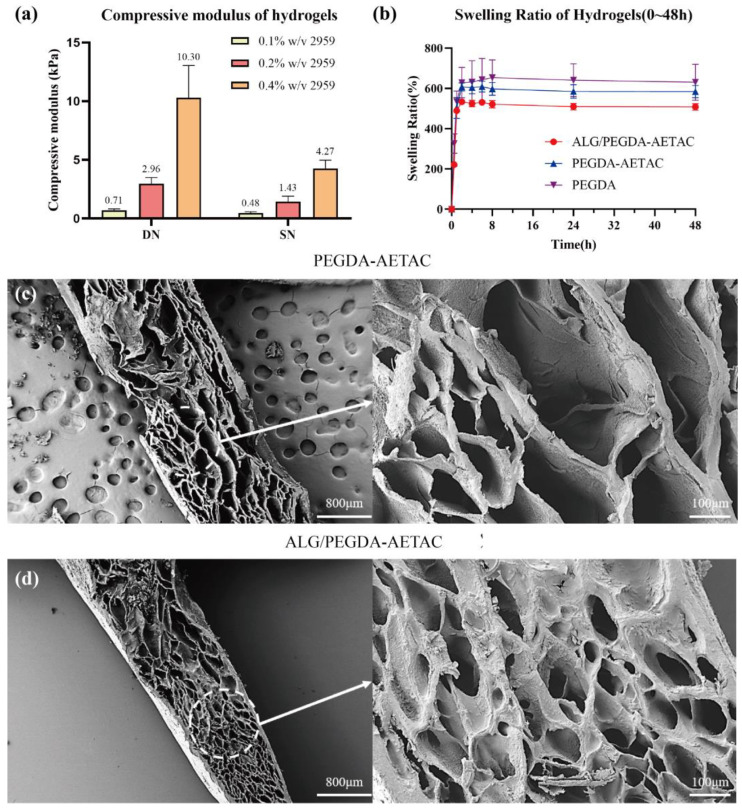
(**a**) Compressive modulus of different hydrogels. (**b**) Swelling ratio of different hydrogels. SEM images of (**c**) PEGDA-AETAC hydrogel and (**d**) ALG/PEGDA-AETAC hydrogel.

The first part of the hydrogel characterization study focused on designing SN and DN hydrogels with adjustable mechanical properties as an initial step in the manufacturing of new hydrogel systems for protein delivery applications. To achieve this goal, the mechanical stiffness of the SN hydrogel was adjusted by changing the concentration of the photoinitiator. This factor affected the crosslink density in the second network, which was necessary for creating a DN structure with adjustable elasticity [36]. Figure 2a shows the compression modulus of the ALG/PEGDA-AETAC DN hydrogel and PEGDA-AETAC SN hydrogel with an Igracure 2959 concentration of 0.1% *w*/*v*, 0.2% *w*/*v*, and 0.4% *w*/*v*. It can be observed that the compressive strength of the ALG/PEGDA-AETAC DN hydrogel was higher than that of the PEGDA-AETAC SN hydrogel. When the concentration of Igracure 2959 was not lower than 0.2% *w*/*v*, the compressive modulus of the ALG/PEGDA-AETAC DN hydrogel was more than twice that of the PEGDA-AETAC SN. Moreover, as the concentration of Igracure 2959 increased, the compressive properties of the hydrogels increased. Since Igracure 2959 has a certain biological toxicity, its concentration should not be too high; therefore, 0.2% *w*/*v* was selected for subsequent experiments [14]. However, a compression modulus of 10 kPa is more than sufficient for the repair of soft tissues in the body. 

In addition to characterizing the mechanical properties, we also detected the swelling ratio, which is also an important evaluation of the hydrogel’s ability to absorb tissue exudates as well as volume stability under wet conditions [37]. Figure 2b shows the change in the swelling rate of the hydrogel within 0–48 h. Initially, the wet weight of the hydrogel changed significantly and reached a peak at approximately 8 h. After 24 h, the wet weight of the hydrogel was stable. The swelling rates of PEGDA, PEGDA-AETAC, and ALG/PEGDA-AETAC hydrogels after 24 h were approximately 639.43%, 587.24%, and 510.35%, respectively, which shows that the swelling rate of the SN hydrogel was always greater than that of the dual-network hydrogels, and the swelling rate of the hydrogels containing AETAC groups was lower than that of the hydrogels without AETAC groups. This may be because when more substances that can form a network are added, the inner network of the hydrogel is denser, which hinders the swelling of the entire structure. In addition, when the hydrogel was stored for 45 days, the quality of the rest of the hydrogels was relatively stable as shown in Figure S1.

Porosity is closely related to the mechanical properties and permeability of the matrix, because it affects the encapsulation of active molecules, the supply of nutrients and oxygen, and the discharge of metabolic waste [38]. As shown in Figure 2c,d, the PEGDA-AETAC SN hydrogel and ALG/PEGDA-AETAC DN hydrogels both had a porous structure. The ALG/PEGDA-AETAC DN hydrogel presented a denser structure with a smaller mesh size owing to the addition of the second network of sodium alginate and calcium ions. The formation of this structure further hinders the release of the drug and plays a key role in the adjustment of the release kinetics.

### 3.3. Biocompatibility

**Figure 3:**
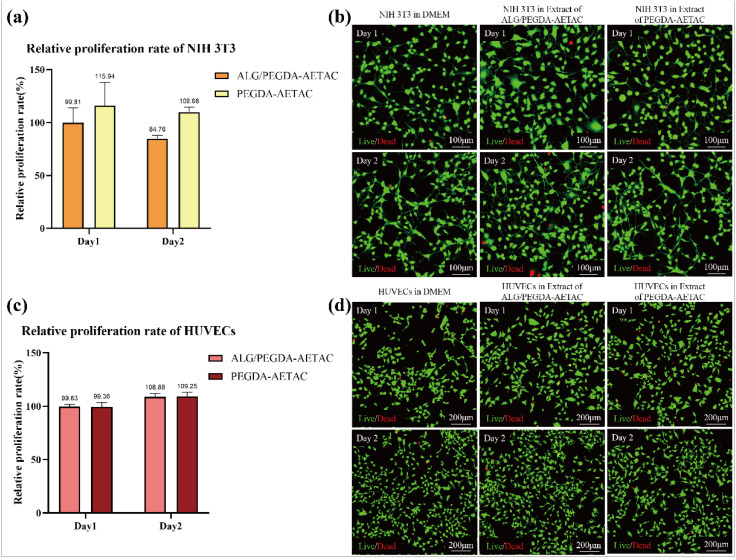
(**a**) The relative proliferation rate of NIH 3T3 cells in the extracts of ALG/PEGDA-AETAC and PEGDA-AETAC. (**b**) Live/dead staining images of NIH 3T3 cells after 24 h and 48 h of culture in culture medium, ALG/PEGDA-AETAC hydrogel extract, and PEGDA-AETAC hydrogel extract. (**c**) The relative proliferation rate of HUVECs in the extracts of ALG/PEGDA-AETAC and PEGDA-AETAC. (**d**) Live/dead staining images of HUVECs after 24 h and 48 h of culture in culture medium, ALG/PEGDA-AETAC hydrogel extract, and PEGDA-AETAC hydrogel extract.

Biocompatibility is a prerequisite for determining whether a hydrogel can be used further. We evaluated the biocompatibility of the new DN hydrogel carrier by studying the toxicity caused by the presence of AETAC. In this study, we evaluated the growth of NIH 3T3 cells and HUVECs in the extracts of SN and DN hydrogels to verify their cytotoxicity. As shown in Figure 3a, the relative proliferation rate of NIH 3T3 cells in the extracts of ALG/PEGDA-AETAC and PEGDA-AETAC at day one and day two showed that the ALG/PEGDA-AETAC hydrogel did not harm cell growth, and the relative proliferation rates of NIH 3T3 cells in the two extracts at day one and day two were 99.8% and 115.9%, respectively. Furthermore, the relative proliferation rate of HUVECs in the extracts of ALG/PEGDA-AETAC and PEGDA-AETAC at day one and day two also showed that the ALG/PEGDA-AETAC hydrogel had little cytotoxicity as shown in Figure 3c, and the relative proliferation rates of HUVECs in the two extracts at day one and day two were 99.63% and 108.88%, respectively. According to the ISO 10993-5:2009 cytotoxicity classification standard, the cytotoxicity level was level one, which is nontoxic [39]. As shown in Figure 3b,d, the live/dead staining images of NIH 3T3 cells and HUVECs cultured for 48 h in culture medium, ALG/PEGDA-AETAC hydrogel extract, and PEGDA-AETAC hydrogel extract indicated that there was no visible difference among the three groups, which proves that the ALG/PEGDA-AETAC hydrogel has no obvious cytotoxicity.

### 3.4. Protein Controlled Release Study

Hydrogels have promising potential as drug carriers in biomedical application [40,41]. Herein, we studied the molecular delivery properties of the DN hydrogel. 

**Figure 4:**
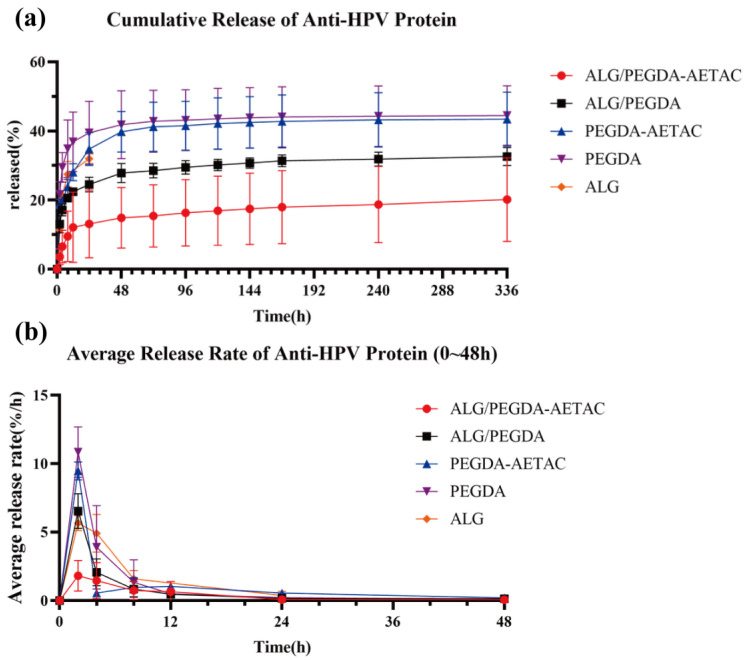
(**a**) Cumulative release of anti-HPV protein. (**b**) Average release rate of anti-HPV protein.

As previously mentioned, a dense structure with a smaller mesh size was formed, which can be used to further adjust the release kinetics. Figure 4a shows the kinetic release curves of the anti-HPV protein in different groups. Since ALG hydrogel degrades after 24 h, the protein release kinetic curve of ALG can only be measured up to 24 h. The remaining hydrogels showed no degradation, so the release amount could be continuously recorded for more than 336 h (14 d), which is a major improvement compared with our previous work [34].

By comparing the protein release kinetic curves of the hydrogels, it was found that the release rate of anti-HPV protein from the hydrogels showed the following two phenomena:

1. The charged group can inhibit the release of drugs with opposite charges.
2. The formation of the DN will greatly hinder the release of drug molecules from the hydrogel.

ALG hydrogel can release approximately 30% of the anti-HPV protein in 24 h. Within 14 days, the PEGDA and PEGDA-AETAC SN hydrogel can achieve approximately 40% release of anti-HPV protein. The presence of the second network reduced the release rate, as shown by the results of the ALG/PEGDA DN and ALG/PEGDA-AETAC DN hydrogel, which achieved approximately 30% and 18% of the release of anti-HPV protein, respectively. Moreover, in the first 48 h, the SN hydrogels showed rapid release of protein, and it can be clearly observed from the curve that the release of protein from the DN hydrogel was gentler.

The average protein release rate curve of the hydrogel is shown in Figure 4b. For both PEGDA-AETAC and PEGDA SN hydrogels, the protein release rate showed a fast release rate within 0–2 h and a sharp drop in the release rate within 2–4 h, which means that the SN hydrogel with PEGDA as the main component does not have a strong ability to hinder the burst release of the anti-HPV protein. After 48 h, the release of anti-HPV proteins from these two types of hydrogels was very low. The addition of AETAC provides the PEGDA-AETAC SN hydrogel with a stronger release control effect on the anti-HPV protein than the PEGDA SN hydrogel. The release rate of anti-HPV protein from the ALG SN hydrogel also showed a gradually decreasing trend, but its degradation rate was too fast to achieve a 14-day long-term release. For ALG/PEGDA-AETAC and ALG/PEGDA DN hydrogels, the initial release rate of the anti-HPV protein was more stable than that of the SN hydrogels. After 48 h, these two types of hydrogels remained relatively stable. Furthermore, the addition of AETAC-charged groups enhanced the controlled release effect of the DN hydrogel on the anti-HPV protein. The cumulative release of ALG/PEGDA-AETAC is about 11.79% at 24 h, and reached approximately 17.66% at 366 h, indicating that the protein still sustained release after 24 h. Initially, some protein in the DN hydrogel was free because there were not enough charged ligands (AETAC) to bind all the protein. Therefore, the protein release rate in the first 24 h was relative fast and the slope of the curve was relatively large. Subsequently, the protein release was mainly achieved by reversible dissociation from the charged ligand (AETAC), which led to the release curve entering a sustained-release phase and produced a smaller slope.

The above experimental phenomena show that the DN hydrogel has a stronger release control effect on the anti-HPV protein than the SN hydrogel. In addition, the addition of the charged group AETAC enhanced the controlled release effect of the hydrogel because the addition of AETAC enhances the affinity of the hydrogel for the negatively charged anti-HPV protein. Therefore, ALG/PEGDA-AETAC had the best controlled release effect on the anti-HPV protein. At the same time, combined with the swelling curves of ALG/PEGDA-AETAC, PEGDA-AETAC, and PEGDA hydrogels, it can be seen that the wet weights of the four types of hydrogels remained relatively stable within 14 days without rapid degradation. Therefore, ALG/PEGDA-AETAC does not rely on degradation to release anti-HPV protein but releases anti-HPV protein through an affinity-controlled release mechanism. Another interesting point is that the effect of AETAC on SN hydrogels is much smaller than that of AETAC on DN hydrogels. One possible explanation is that the introduction of negatively charged sodium alginate in the double network is more beneficial for maintaining AETAC in the hydrogel. In a single network, AETAC may be free from the hydrogel together with the anti-HPV protein because it is not well-fixed in the PEGDA molecular network.

### 3.5. Printability

**Figure 5:**
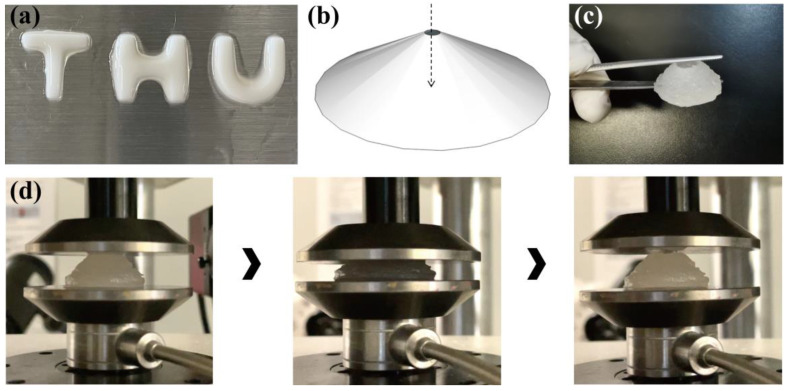
(**a**) “THU” model printed by DLP. (**b**) CAD file of a cone with hollow structure. (**c**) A cone with hollow structure printed by DLP. (**d**) Compression and rebound of the printed structure.

Figure 5a shows the result of the “THU” model printed by DLP under the 3 s exposure time of each layer. Each letter has a length and width of 1.5 mm, height of 4 mm, and slice thickness of 0.1 mm. The printed structure has a clear outline and no internal defects, which proves the feasibility of printing the ALG/PEGDA-AETAC DN hydrogel model using DLP. Given the feasibility of printing, a cone with a hollow structure (shown in Figure 5b) was printed to test the printability of a complex structure, and it can be observed that the ALG/PEGDA-AETAC DN hydrogel can realize accurate shaping according to the preset model, even for a more complex structure, as shown in Figure 5c. The above structure can be utilized as a tissue patch after cervical cancer conization surgery. Moreover, it can be observed from Figure 5d and Video S1 that the printed structure can recover the initial morphology after compression, indicating a good stretchability with great potential. 

## 4. Conclusions

In this study, we developed a DN hydrogel, the main components of which were PEGDA, sodium alginate, and the positively charged monomer AETAC. By adjusting the concentration of the photoinitiator, DN hydrogels with different compressive moduli in the range of 0.71–10.3 kPa were fabricated, which match the modulus of human soft tissues in the body. The SN and DN hydrogels were also found to support cell growth according to cytotoxicity tests with the result of over 100% relative proliferation rate of HUVECs. Based on the affinity-controlled delivery mechanism inspired by the controlled release found in the ECM, charged groups can inhibit protein burst release and achieve prolonged release (over 14 days) with the help of electrostatic interaction between protein drugs with opposite charges. Moreover, the denser network also contributed to the protein sustained release compared to the SN hydrogels. In addition, the material can be printed by DLP 3D printing technique to produce a complex structure with high elasticity, which has great application potential in tissue engineering.

## Figures and Tables

**Table 1:** Ingredients in hydrogels loaded with anti-HPV protein.

Group	ALG (wt%)	Irgacure 2959 (*w*/*v*)	PEGDA (wt%)	AETAC (wt%)	CaCl_2_ (Mm)	Anti-HPV Protein (mg/mL)
PEGDA	-	0.2%	20%	-	-	5
PEGDA-AETAC	-	0.2%	20%	5%	-	5
ALG	2.5%	-	-	-	25	5
ALG/PEGDA	2.5%	0.2%	20%	-	25	5
ALG/PEGDA-AETAC	2.5%	0.2%	20%	5%	25	5

## Data Availability

Not applicable.

## Supplementary Materials

The following are available online at https://www.mdpi.com/article/10.3390/biomedicines9101298/s1, Figure S1: Swelling ratio of different hydrogels (0-45D), Video S1: compression and recovery of cervix implant.
