# Supplementary figures and images for: Affinity-Controlled Double-Network Hydrogel Facilitates Long-Term Release of Anti-Human Papillomavirus Protein
Source: Biomedicines. 2021 Sep 23;9(10):1298. doi: 10.3390/biomedicines9101298 (PMC8533454; doi:10.3390/biomedicines9101298)

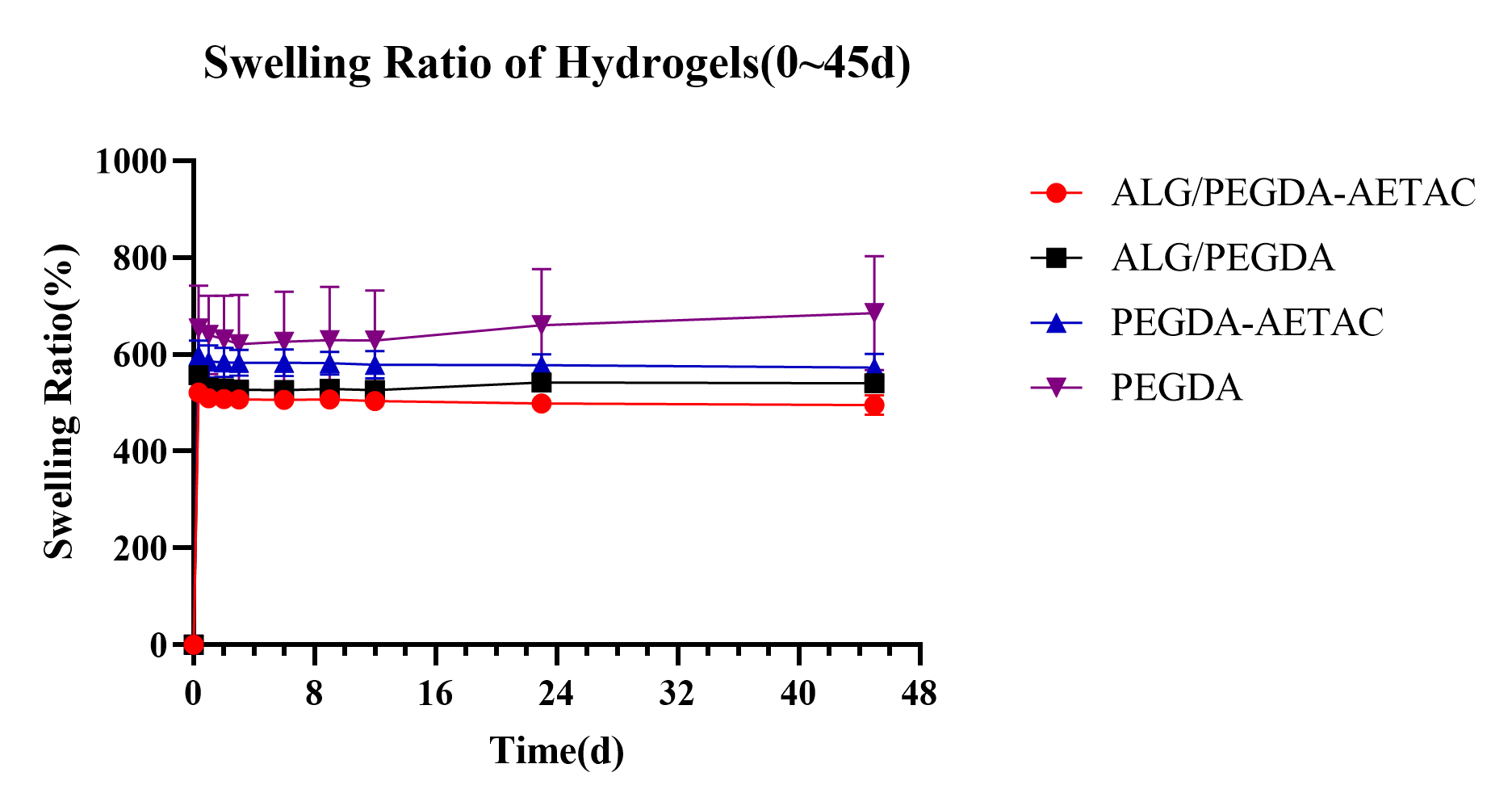

Supplement: Supplementary file 1 [file biomedicines-09-01298-s001.zip › Figure S1 swelling ratio 0~45d.tif]
